# Efficacy and Safety of Zilebesiran for the Management of Hypertension: An Updated Systematic Review and Meta-Analysis of Randomised Controlled Trials

**DOI:** 10.7759/cureus.92446

**Published:** 2025-09-16

**Authors:** Muhammad Raza, Chaudhry Zaid Riaz, Eymaan Riaz Chaudhry, Misha Jannat, Abdul Raffeh Basit, Ali Jamshed Qureshi, Muhammad Hassan Qureshi, Muhammad Waleed Ahmed

**Affiliations:** 1 Medicine, Combined Military Hospital (CMH) Lahore Medical College and Institute of Dentistry, Lahore, PAK; 2 Internal Medicine, Combined Military Hospital, Lahore, PAK; 3 Internal Medicine, Afzal Hospital, Jhelum, PAK; 4 Internal Medicine, NHS England Education East Midlands, Nottingham, GBR; 5 Medicine, Jinnah Sindh Medical University, Karachi, PAK; 6 Global Health and Public Policy, Queen Mary University of London, London, GBR; 7 Internal Medicine, UCHealth Parkview Hospital, Pueblo, USA; 8 Public Health, Anglia Ruskin University, Cambridge, GBR; 9 Emergency Medicine, Combined Military Hospital, Lahore, PAK

**Keywords:** angiotensinogen, antihypertensive drugs, high blood pressure, hypertension, rna interference agent, zilebesiran

## Abstract

Zilebesiran is a novel RNA interference (RNAi) therapeutic agent that reduces the production of hepatic angiotensinogen, showing promising results in the management of hypertension. However, a comprehensive assessment of its efficacy and safety is still required. This meta-analysis included four randomised controlled trials (RCTs) with 1,037 participants across the United States, Europe, and Asia, assessing single and repeat subcutaneous doses, mostly in patients on background antihypertensive therapy. The primary outcomes were change from baseline in 24-hour ambulatory and mean office blood pressure (BP) outcomes (results expressed in mmHg), adverse events, and markers of renal function. An extensive search was conducted across five electronic databases to identify RCTs assessing the clinical impact of Zilebesiran. Review Manager version 5.4 (RevMan 5.4; The Cochrane Collaboration, London, England, UK) was used to analyse data under a random-effects model, and results were reported using mean difference (MD), standardised mean difference (SMD), and odds ratio (OR) with 95% confidence intervals (CI). Zilebesiran resulted in a significant reduction of 24-hour ambulatory systolic blood pressure (SBP) at 12 weeks (MD -11.90; 95% CI: -13.42, -10.38; p-value <0.00001) and 24 weeks (MD -9.05; 95% CI: -10.37, -7.73; p-value <0.00001). Mean office SBP was also significantly lowered at 12 weeks (MD -10.68; 95% CI: -12.21, -9.14; p-value <0.00001) and 24 weeks (MD -8.49; 95% CI: -9.70, -7.28; p-value <0.00001). Meaningful reductions in 24-hour ambulatory diastolic blood pressure (DBP) were observed at 12 weeks (MD -8.67; 95% CI: -9.89, -7.47; p-value <0.00001) and 24 weeks (MD -7.90; 95% CI: -9.12, -6.68; p-value <0.00001). Zilebesiran was associated with a modest increase in total adverse events (OR 1.37; 95% CI: 1.06, 1.76; p-value =0.01), but showed no significant difference in severe adverse events compared to placebo (OR 0.75; 95% CI: 0.36, 1.57; p-value =0.45). Changes in serum creatinine (SMD -0.01; 95% CI: -0.16, 0.13; p-value =0.20) and eGFR (estimated glomerular filtration rate) levels (SMD -0.06; 95% CI: -0.21, 0.08; p-value =0.39) were also not statistically significant. This meta-analysis confirms that Zilebesiran is consistently associated with significant reductions in both ambulatory and office blood pressure over 12 to 24 weeks, with an acceptable safety profile. Further trials are needed to evaluate the efficacy, dosing, and safety outcomes of Zilebesiran across diverse hypertensive populations.

## Introduction and background

High blood pressure, commonly known as ‘hypertension’, is a consistently high force of blood pushing against the arterial walls, which over time damages them and leads to serious complications. It is commonly referred to as a ‘silent killer’ because it remains asymptomatic for long periods. Hypertension affects 116 million adults in the United States alone, contributing to more than 670,000 deaths in 2020 [1]. Worldwide, it affects almost 1.28 billion adults aged 30-79, of whom only one in five has it under control. Disease prevalence varies by region, with the WHO African Region reporting the highest rates (27%) compared to the lowest in the WHO Region of the Americas (18%), highlighting significant disparities. Hypertension remains one of the leading global causes of premature death, and a key WHO target is to reduce its prevalence by 33% between 2010 and 2030 [2]. The risk of having hypertension increases with age, stress, being overweight, smoking, increased consumption of sodium, alcohol consumption and leading a sedentary lifestyle [3]. Unless specified otherwise, prevalence figures in this review refer to diagnosed hypertension, while control rates indicate those meeting guideline-recommended blood pressure thresholds under treatment.

The current treatment strategy for hypertension involves lifestyle modifications and the use of oral antihypertensive agents, such as angiotensin-converting enzyme inhibitors (ACEIs), angiotensin receptor blockers (ARBs), calcium channel blockers (CCBs), diuretics, and beta blockers [4]. Despite the availability of a wide range of oral antihypertensive agents, only one in five individuals with hypertension has it under control [2]. There are several reasons for this gap, including resistant hypertension, where blood pressure remains uncontrolled despite the use of at least three different antihypertensive classes (including a diuretic). In addition, many agents are associated with side effects, which can further reduce adherence. Beyond dosing complexity, medication non-compliance and polypharmacy, real-world adherence data show that up to 50% of patients discontinue therapy within the first year, reflecting the burden of long-term daily medication intake and adverse effects [5]. This lays emphasis on the necessity of formulating and investigating novel treatment methods with a simple dosing regimen, which can be used as an add-on therapy alongside oral antihypertensive agents [5].

The renin angiotensin aldosterone system (RAAS) plays a pivotal role in the pathogenesis of hypertension. An investigational agent, Zilebesiran, is an RNA interference agent made up of a small interfering RNA (siRNA) covalently linked to a N-acetylgalactosamine (GaINAc) ligand. It works by binding to the hepatic asialoglycoprotein receptor with high affinity. It reduces the production of angiotensinogen by reducing hepatic angiotensinogen messenger RNA (mRNA) levels, exerting its therapeutic role in the management of hypertension [6]. All angiotensin peptides are derived from angiotensinogen. So, in theory, RAAS inhibition using this approach can limit the role of angiotensin peptides in the pathophysiology of hypertension. Zilebesiran reduces hepatic angiotensinogen production, thereby lowering all downstream angiotensin peptides, which may offer added value in resistant or inadequately controlled hypertension [7].

Zilebesiran is a subcutaneously administered agent, given every 12 to 24 weeks, with a prolonged duration of action, offering the potential of sustained reduction in blood pressure for weeks. Early-phase clinical trials have demonstrated promising pharmacokinetics and pharmacodynamics, with a rapid onset of angiotensinogen suppression maintained for 12 to 24 weeks after a single subcutaneous injection [6]. Dose-ranging studies show that higher doses produce greater and more durable reductions in circulating angiotensinogen and blood pressure, with effects sustained for up to six months in some cohorts. Importantly, Zilebesiran is being investigated both as monotherapy and as an add-on to standard oral antihypertensive regimens, including in patients with high cardiovascular risk and resistant hypertension. This long-acting profile, combined with the potential to reduce adherence-related treatment failures, makes Zilebesiran an attractive candidate for addressing key unmet needs in hypertension management.

Zilebesiran has been studied both as monotherapy and as an add-on to standard oral antihypertensive therapy. Early phase trials, including KARDIA-1, tested dose-ranging monotherapy, while later studies, such as KARDIA-2, evaluated its use in patients with uncontrolled or high-risk hypertension already receiving background therapy. Together, the three available randomised controlled trials (RCTs) provide key evidence: dose-response and duration data, efficacy and safety versus placebo across ambulatory and office blood pressure endpoints, and results for both monotherapy and add-on use. KARDIA-2 is particularly important as it enrolled a larger and more diverse population, tested Zilebesiran alongside standard therapy to reflect real-world practice, and included longer follow-up, offering the most current data on sustained blood pressure effects and safety.

We conducted this updated systematic review and meta-analysis to consolidate the most recent and comprehensive evidence on the efficacy and safety of Zilebesiran in lowering blood pressure in patients with hypertension. Existing reviews on Zilebesiran are scarce and limited in scope, typically including only two RCTs. In contrast, this meta-analysis incorporates three high-quality RCTs, including the most recent KARDIA-2 trial results, making this the most up-to-date and complete analysis to date. We extracted and analysed all available primary and secondary endpoints, including adverse event profiles across these trials, to ensure a thorough evaluation of both efficacy and safety outcomes. Given the clinical interest in Zilebesiran, due to its unique mechanism of action and the potential for sustained blood pressure control with infrequent dosing, there was a clear need to reassess its efficacy and safety profile using the latest trial data. Our aim is to provide clinicians and researchers with clear guidance by synthesising the latest evidence and evaluating the therapeutic potential of Zilebesiran in the management of hypertension.

## Review

Methods

The effectiveness and safety of Zilebesiran in lowering blood pressure among patients with hypertension, as demonstrated by the quantitative analysis of data from completed RCTs, is the research question that serves as the foundation of this systematic review and meta-analysis. This meta-analysis was completed in accordance with the established PRISMA (Preferred Reporting Items for Systematic Reviews and Meta-Analyses) guidelines [8].

Data Sources and Search Strategy

Five electronic research databases were used to carry out this search, including PubMed, Cochrane Central, Science Direct, Google Scholar and clinicaltrials.gov. The search was conducted using a combination of MeSH terms and keywords: “Zilebesiran” OR “ALN-AGT01” AND “hypertension” OR “HTN” OR “high blood pressure" AND “randomised controlled trial” OR “RCT” OR “trial”. Boolean operators (AND/OR) and database-specific filters were applied for the presence of these keywords in the title of the article, abstract, or amongst the terms specified by the author in most databases. This search was last carried out in May 2025. No restrictions were placed on the year of publication. Reference lists of included articles were screened manually.

Inclusion and Exclusion Criteria

Table 1 illustrates the inclusion and exclusion criteria for this meta-analysis.

**Table 1:** Inclusion and exclusion criteria

Study	Inclusion criteria	Exclusion criteria
Study design	Only randomised controlled trials (RCTs) were included. Trials could be either double-blinded or single-blinded, provided the randomisation process was clearly described.	Observational studies, case series, case reports, reviews, editorials, and preclinical studies were excluded.
Population	Adult patients (≥18 years) with pre-existing hypertension.	Paediatric population
Intervention	Studies in which Zilebesiran was administered as a monotherapy or in combination with standard antihypertensive agents.	Studies that combined Zilebesiran with agents not relevant to standard hypertension management.
Comparator	Placebo, no treatment, or standard antihypertensive therapy.	Studies without a control/comparator group.
Study outcomes	Change in systolic or diastolic blood pressurefrom baseline, measured by office or ambulatory blood pressure monitoring.	Studies not reporting relevant outcomes related to Zilebesiran, or lacking extractable quantitative data were excluded.
Publication status	Peer-reviewed articles published in indexed journals up to May 31, 2025.	Non-peer-reviewed publications, abstracts without full texts, and grey literature.
Language	Studies reported in the English language.	Studies not reported in English language.

Ethical Approval and Quality Assessment

As this study involved the collection and analysis of data from previously published RCTs and no new patient data was collected, ethical approval was not required. The risk of bias assessment was carried out by two reviewers using the Risk of Bias (RoB) 2.0 tool for randomised trials [9]. This research was conducted in a transparent and systematic manner, following all relevant protocols outlined in the PRISMA guidelines for conducting systematic reviews and meta-analyses [8].

Data Extraction

Extraction of data was carried out by two reviewers independently to eliminate the risk of bias. Discrepancies were resolved by consensus; a third reviewer arbitrated unresolved disagreements. Microsoft Excel (Microsoft Corp., Redmond, WA, USA) was used to help in data retrieval. The first step of data extraction included study characteristics and patient demographics. Data for all primary and secondary endpoints reported in each of the included studies were extracted into an Excel sheet. After careful review and consideration, study outcomes were structured to ensure they were common to at least two of the included studies. Units of measurement for study outcomes were converted where necessary to standardise effect estimates for inclusion in this meta-analysis. When standard deviation (SD) was not directly reported, it was derived from the reported 95% confidence interval (CI) using the Meta-Analysis Accelerator tool for statistical data conversion accessed online [10].

Statistical Analysis

RevMan version 5.4 (The Cochrane Collaboration, London, England, UK) was used to perform all statistical analyses. Mean difference (MD) was used for continuous outcomes, including ambulatory and mean office blood pressure monitoring, while standardised mean difference (SMD) was applied for serum creatinine and estimated glomerular filtration rate (eGFR) levels due to variation in units of measurement across studies. For dichotomous outcomes, including the number of total and severe adverse events, the odds ratio (OR) was used. All outcomes were reported with corresponding 95% confidence intervals (CI). The I^2^ value was used to report heterogeneity among studies and classified as: low (<25%), moderate (25-50%), and high (>50%). Random-effects models were applied as heterogeneity was often high, and a p-value less than 0.05 was considered statistically significant. Where appropriate, subgroup analyses were reported to explore potential sources of heterogeneity and assess the consistency of findings across studies.

Results

Study Selection

**Figure 1:**
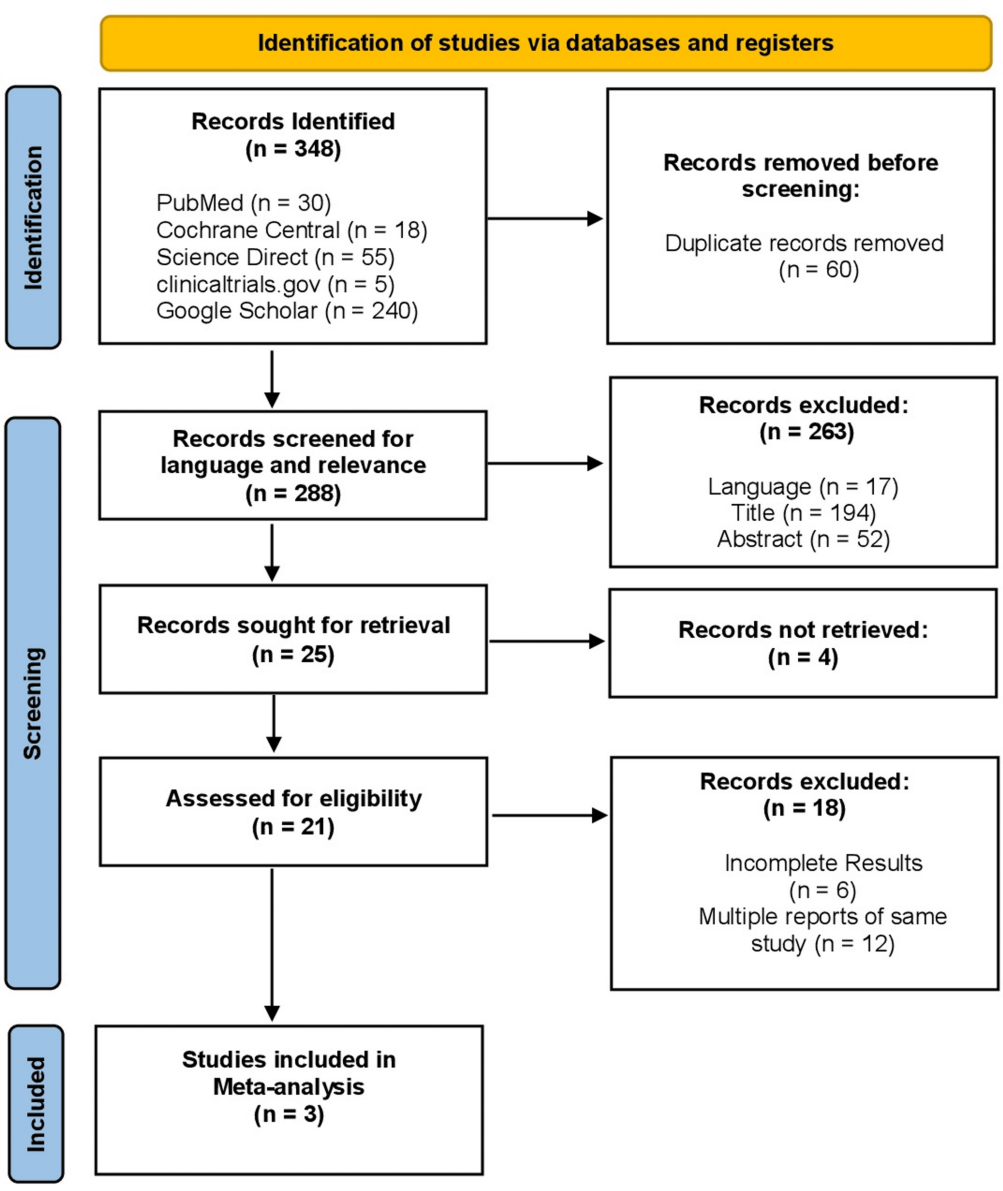
PRISMA flow diagram PRISMA: Preferred Reporting Items for Systematic Reviews and Meta-Analyses [8]

The search results from all databases were screened for relevance, and duplicate records were removed using EndNote Online Classic (Clarivate, Pennsylvania, USA). The study selection process is illustrated in the PRISMA flow diagram (Figure 1) [8]. A total of 348 records were identified after searching the five selected databases. After removing 60 duplicate entries, the remaining 288 records were screened for relevance. These records were assessed for relevance independently by two reviewers based on their titles and abstracts. A total of 263 records were excluded, including 17 that were not in English, 194 had irrelevant titles, and 52 were excluded based on their abstract content. This resulted in 25 full-text articles being selected for retrieval, of which four could not be accessed. These four full-text articles were inaccessible despite attempts to obtain them through institutional library services. The authors were also contacted, but no replies were received; therefore, these studies were excluded. The remaining 21 articles were retrieved in full and assessed comprehensively for eligibility. Eighteen of these were excluded; six due to reporting of RCTs still underway with incomplete results, and 12 were multiple reports of the same RCTs already included in this meta-analysis (multiple reports of the same trial existed, the most comprehensive publication was selected and overlapping cohorts were excluded to avoid duplication).

Study Characteristics

Three RCTs were shortlisted after screening and included in this meta-analysis [11-13]. All three studies were placebo-controlled and followed a double-blind design, at least for the major portion of the study. The total pooled sample size was 1147, with a combined mean age of 57.5 years. Around 45% participants were female individuals, and common co-morbidities included type 2 diabetes, obesity, and dyslipidaemia. The included trials examined a wide range of Zilebesiran doses administered subcutaneously at varying intervals, and in some cases, in combination with other antihypertensive agents. The primary outcome across studies was the reduction in ambulatory systolic blood pressure (SBP), assessed at both 12 and 24 weeks, with results that varied by dosage and treatment groups. Secondary outcomes included changes in mean office SBP, ambulatory diastolic blood pressure (DBP), number of adverse events and markers of renal function such as serum creatinine and eGFR. Detailed characteristics of the included studies are summarised in Table 2.

**Table 2:** Characteristics of included studies SBP: systolic blood pressure [11-13]

Study	Journal	Study design	Country	Sample size	Age (Mean ± SD)	Dosage	Ambulatory SBP reduction at 12 weeks	Ambulatory SBP reduction at 24 weeks
Desai et al. (2023) [11]	The New England Journal of Medicine	Phase 1, randomised, double-blind (Parts A & B), and open-label (Part E), placebo-controlled trial	United Kingdom	107	53.5 ±7.5	Part A:		
						200 mg	-15.0 mmHg	-12.5 mmHg
						400 mg	-13.3 mmHg	-9.3 mmHg
						800 mg	-16.9 mmHg	-22.5 mmHg
						Part B:		
						Zilebesiran 800 mg + high salt intake	-	-
						Part E:		
						Zilebesiran 800 mg	-	-
						Zilebesiran 800 mg + Irbesartan 300 mg	-	-
Bakris et al. (2024) [12]	The Journal of the American Medical Association (JAMA)	Phase 2, randomised, double-blind, placebo-controlled trial	Canada, Ukraine, UK, USA	377	56.8 ± 10.6	150 mg SC every 6 months	-7.3 mmHg	-6.5 mmHg
						300 mg SC every 6 months	-10.0 mmHg	-9.5. mmHg
						300 mg SC every 3 months	-10.0 mmHg	-9.9 mmHg
						600 mg SC every 6 months	-8.9 mmHg	-9.6 mmHg
Desai et al. (2025) [13]	The Journal of the American Medical Association (JAMA)	Phase 2, randomised, prospective, double-blind, placebo-controlled trial	USA, Canada, UK, Germany, Poland, Estonia, Latvia, Lithuania	663	58.5 ±10.3	Zilebesiran 600 mg + Indapamide 2.5 mg	−15.7 mmHg	−15.6 mmHg
						Zilebesiran 600 mg + Amlodipine 5 mg	−10.5 mmHg	−9.7 mmHg
						Zilebesiran 600 mg + Olmesartan 40 mg	−7.7 mmHg	−7.6 mmHg

Risk of Bias Assessment

**Figure 2:**
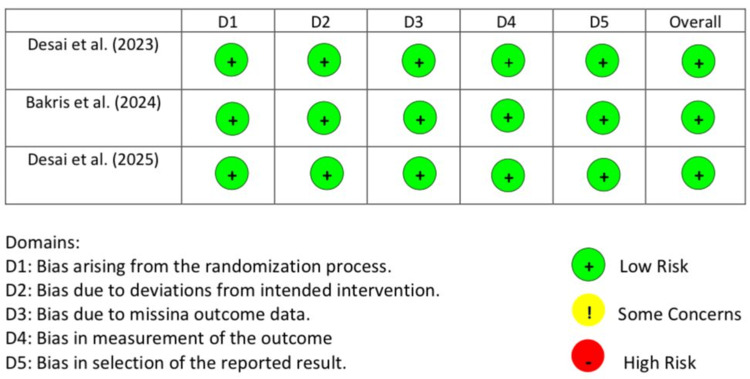
Risk of bias assessment [11-13]

The revised Cochrane ‘Risk of Bias’ tool for randomised controlled trials (RoB 2.0) was used to assess the risk of bias in the included RCTs [14]. Two reviewers independently performed the assessment, with discrepancies resolved by consensus. Results are presented across five domains, including randomisation process, deviations from intended interventions, missing outcome data, measurement of outcomes, and selection of reported results. All three of the included RCTs were reported as high-quality studies with low risk of bias. Figure 2 below shows the results of bias assessment across all the assessed domains. Although most parts of the included studies were double-blinded, we noted potential performance bias in the partially open-label Part E of Desai et al. (2023) [11]. This was acknowledged but did not materially alter pooled estimates in the analyses.

Synthesis of Results

24-hour ambulatory systolic blood pressure (SBP) at 12 weeks: A statistically significant reduction in 24-hour ambulatory SBP at 12 weeks was observed in the group given Zilebesiran in comparison to placebo (MD -11.90; 95% CI: -13.42, -10.38). These values represent weighted pooled estimates across studies. A high level of heterogeneity was reported amongst studies (I²=72%).

**Figure 3:**
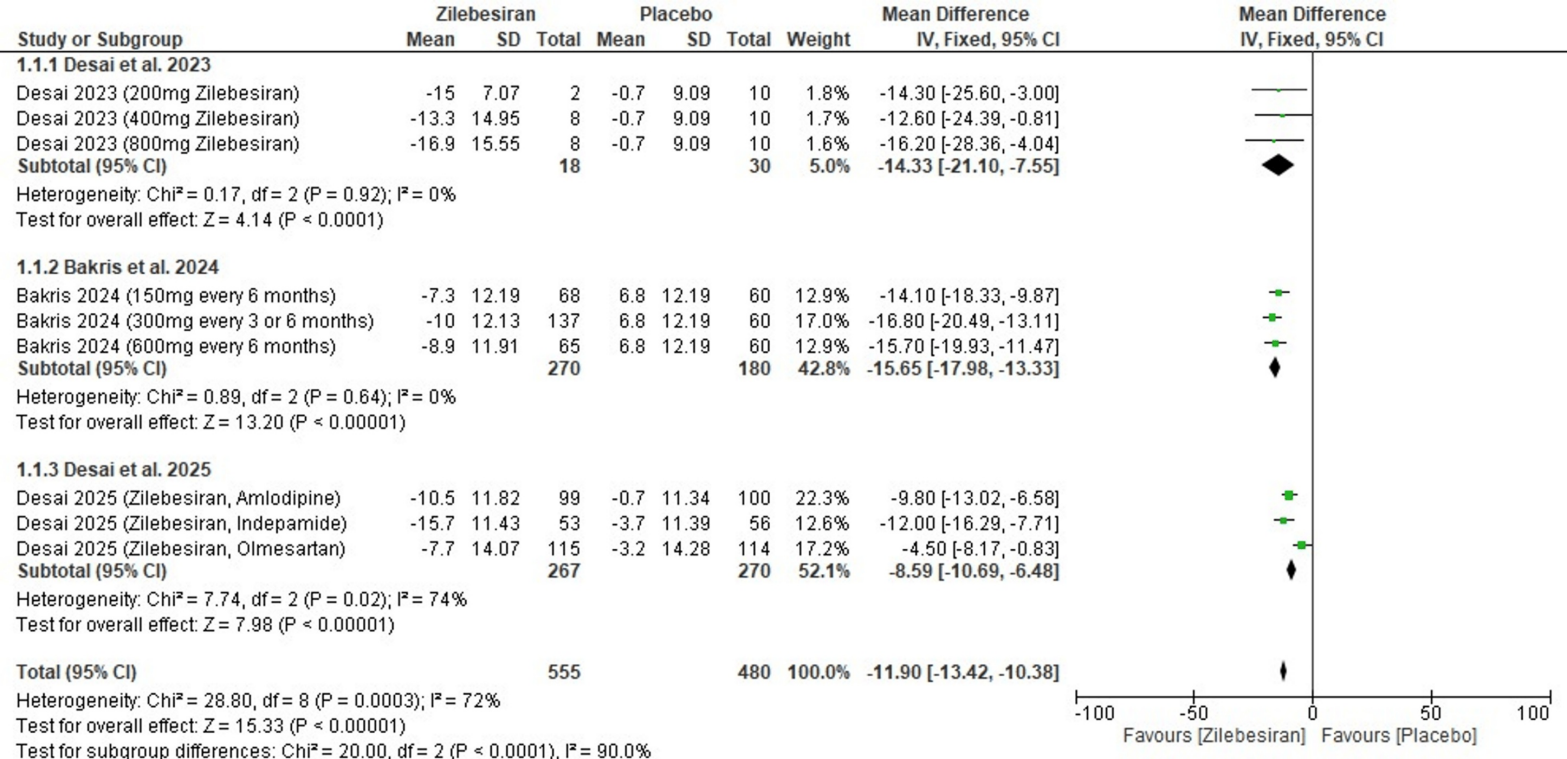
Forest plot for change from baseline in 24-hr ambulatory systolic blood pressure (SBP) at 12 weeks [11-13]

In sub-group analysis, Desai et al. (2023) [11] and Bakris et al. (2024) [12] showed greater ambulatory SBP reductions with pooled mean differences of -14.33 and -15.65 mmHg, respectively, and no observed heterogeneity, suggesting consistency in their findings. In contrast, the study by Desai et al. (2025) [13], which evaluated Zilebesiran in combination with other antihypertensives, reported a smaller but still significant reduction in ambulatory SBP (MD -8.59 mmHg) with moderate heterogeneity. The overall pooled analysis demonstrated a highly significant effect, indicating strong evidence for a reduction in 24-hour ambulatory SBP (Z=15.33; p-value <0.00001). Figure 3 illustrates the forest plot outlining these findings.

24-hour ambulatory systolic blood pressure (SBP) at 24 weeks: Zilebesiran was associated with a significant reduction in 24-hour ambulatory SBP at 24 weeks compared to placebo (MD -9.05; 95% CI: -10.37, -7.73). A high level of heterogeneity was observed across the included studies, indicating variability in effect sizes (I²=82%).

**Figure 4:**
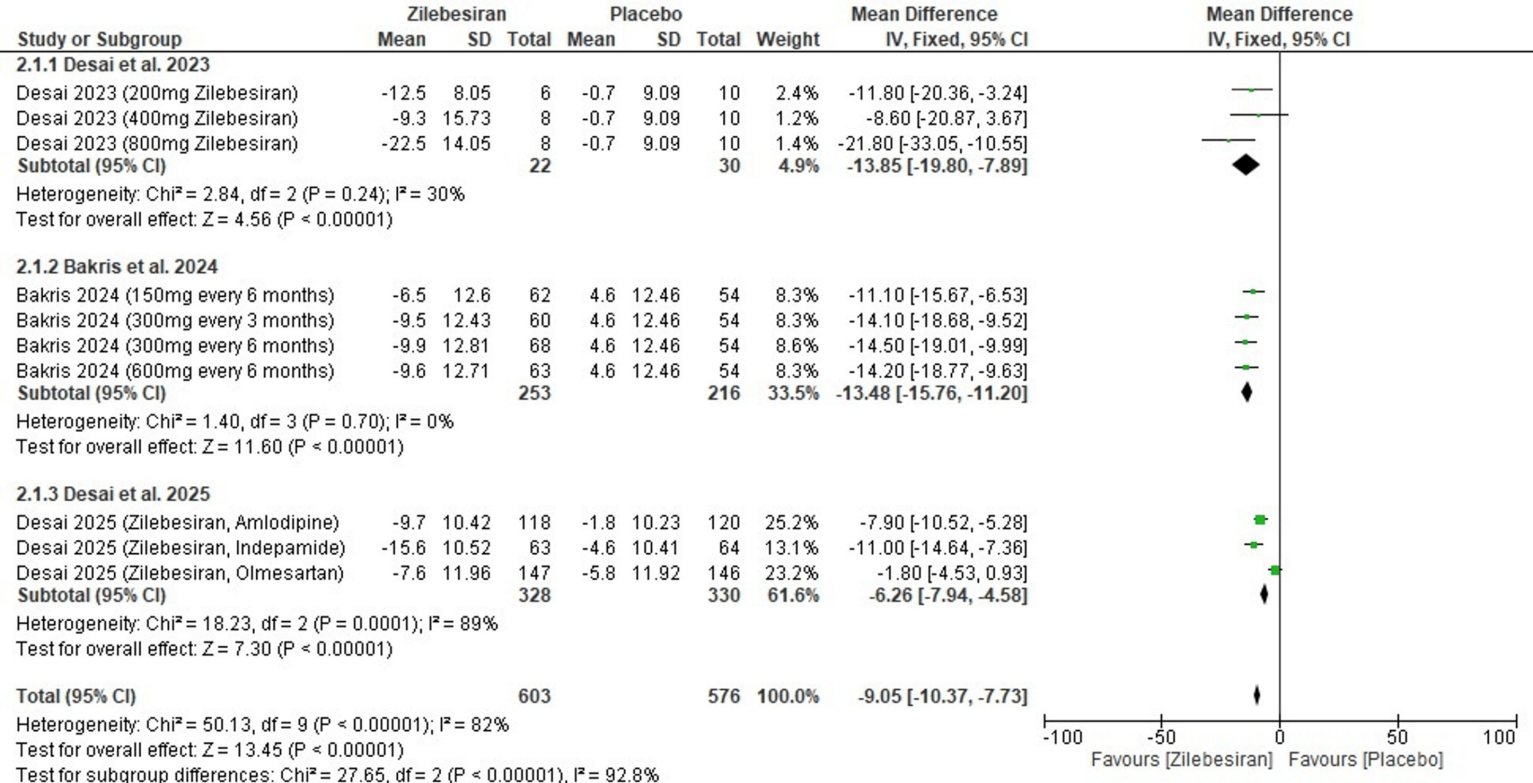
Forest plot for change from baseline in 24-hr ambulatory systolic blood pressure (SBP) at 24 weeks [11-13]

In subgroup analysis, Desai et al. (2023) [11] and Bakris et al. (2024) [12] showed similar significant ambulatory SBP reductions with pooled mean differences of -13.85 and -13.48 mmHg, respectively, and low to no heterogeneity (I²=30% and 0%), while Desai et al. (2025) [13] reported a smaller effect (MD -6.26 mmHg) with high heterogeneity (I²=89%) indicating variability likely due to the influence of combination with other medications. The overall effect confirmed that the reduction in ambulatory SBP with Zilebesiran is highly statistically significant (Z=13.45; p-value <0.00001). These results are illustrated in the forest plot shown in Figure 4.

Mean office systolic blood pressure (SBP) at 12 weeks: Zilebesiran was associated with a significant reduction in mean office SBP at 12 weeks compared to placebo (MD -10.68; 95% CI: -12.21, -9.14). A considerably high level of heterogeneity was observed among the included studies (I²=75%).

**Figure 5:**
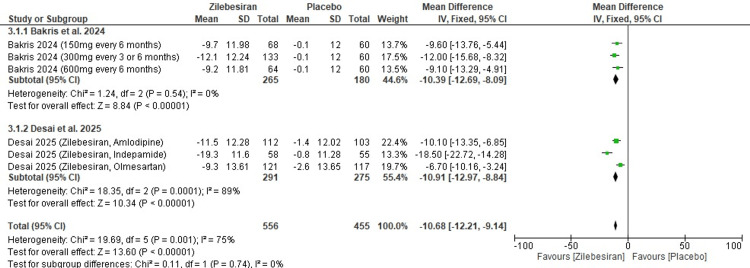
Forest plot for change from baseline in mean office systolic blood pressure (SBP) at 12 weeks [12,13]

In subgroup analysis, Bakris et al. (2024) [12] evaluated different doses of Zilebesiran administered every three to six months and reported a consistent mean office SBP reduction (MD -10.39 mmHg; 95% CI: -12.69 to -8.09) with no heterogeneity, indicating uniform findings across dosing regimens. In contrast, Desai et al. (2025) [13], which assessed Zilebesiran in combination with other antihypertensive agents, showed a comparable mean reduction (MD -10.91 mmHg; 95% CI: -12.97 to -8.84) but with high heterogeneity. Despite this heterogeneity, the effect was consistently in favour of Zilebesiran across all subgroups. The pooled analysis confirmed the significance of the observed mean office SBP reduction (Z=13.60; p-value <0.00001), as presented in Figure 5.

**Figure 6:**
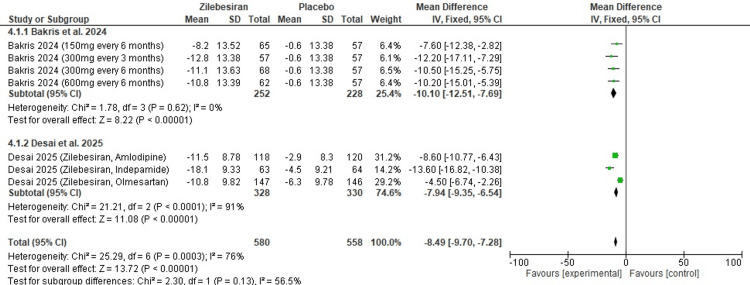
Forest plot for change from baseline in mean office systolic blood pressure (SBP) at 24 weeks [12,13]

Mean office systolic blood pressure (SBP) at 24 weeks: Zilebesiran resulted in a significant reduction in mean office SBP at 24 weeks in comparison to placebo (MD -8.49; 95% CI: -9.70, -7.28), as illustrated in Figure 6. A high level of heterogeneity was observed among the included studies (I²=76%).

In subgroup analysis, Bakris et al. (2024) [12] evaluated different doses of Zilebesiran administered every three to six months and reported a consistent mean office SBP reduction (MD -10.10 mmHg; 95% CI: -12.51 to -7.69) with no heterogeneity. In comparison, Desai et al. (2025) [13] assessed Zilebesiran in combination with other antihypertensive agents, showing a slightly smaller but still significant reduction (MD -7.94 mmHg; 95% CI: -9.35 to -6.54) accompanied by high heterogeneity (I²=91%), likely due to differences in background therapy. All results were consistently in favour of Zilebesiran across all subgroups; the overall effect was highly statistically significant (Z=13.72; p-value <0.00001).

**Figure 7:**
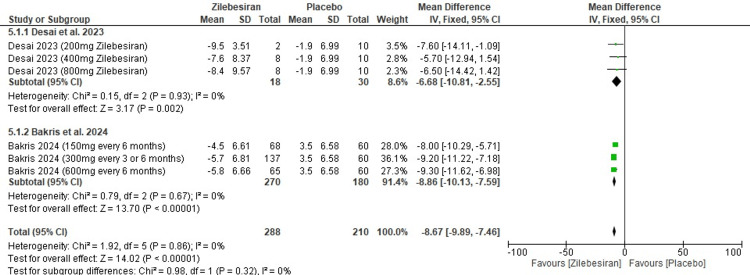
Forest plot for change from baseline in 24-hr ambulatory diastolic blood pressure (DBP) at 12 weeks [11,12]

24-hour ambulatory diastolic blood pressure (DBP) at 12 weeks: Zilebesiran significantly lowered 24-hour ambulatory DBP at 12 weeks compared to placebo (MD -8.67; 95% CI: -9.89, -7.47), based on a random-effects model with no observed heterogeneity across studies (I²=0%). In subgroup analysis, Desai et al. (2023) [11] and Bakris et al. (2024) [12] both reported significant reductions with consistent findings and no heterogeneity. The overall effect was highly statistically significant (Z=14.02; p-value <0.00001). These results are illustrated in the forest plot shown in Figure 7.

**Figure 8:**
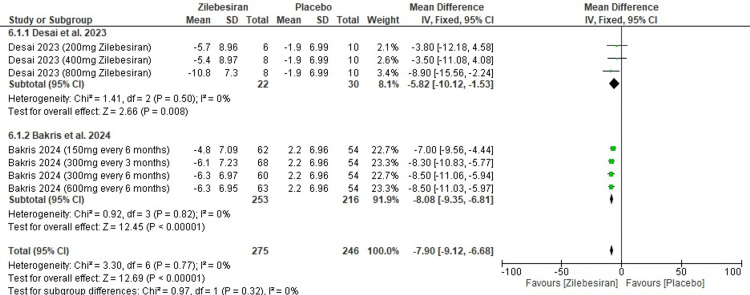
Forest plot for change from baseline in 24-hr ambulatory diastolic blood pressure (DBP) at 24 weeks [11,12]

24-hour ambulatory diastolic blood pressure (DBP) at 24 weeks: Zilebesiran led to a significant decrease in 24-hour ambulatory DBP at 24 weeks compared to placebo (MD -7.90; 95% CI: -9.12, -6.68), also using a random-effects model with no heterogeneity observed (I²=0%). In subgroup analysis, Desai et al. (2023) [11] and Bakris et al. (2024) [12] both reported meaningful ambulatory DBP reductions with consistent outcomes and no variation between studies. The overall effect was highly significant (Z=12.69; p-value <0.00001). These findings are depicted in the forest plot presented in Figure 8.

**Figure 9:**
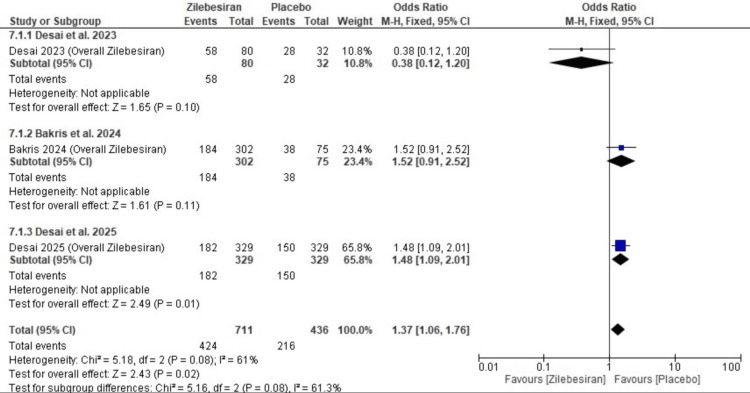
Forest plot for total adverse events comparing Zilebesiran and placebo [11-13]

Total adverse events: Zilebesiran was associated with a higher risk of total adverse events compared to placebo (OR 1.37; 95% CI: 1.06, 1.76), with moderate heterogeneity observed (I²=61%). Subgroup analysis showed variable results; Desai et al. (2023) [12] reported fewer adverse events (OR 0.38; 95% CI: 0.12-1.20), while Bakris et al. (2024) [12] and Desai et al. (2025) [13] reported more (OR 1.52 and OR 1.48, respectively). A detailed overview of these findings is provided in the forest plot shown in Figure 9.

**Figure 10:**
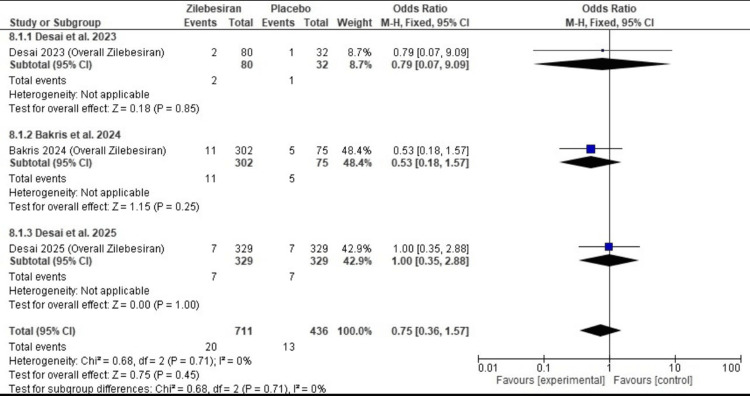
Forest plot for severe adverse events comparing Zilebesiran and placebo [11-13]

Severe adverse events: There was no statistically significant difference in the incidence of severe adverse events between the Zilebesiran and placebo groups (OR 0.75; 95% CI: 0.36, 1.57). No heterogeneity was observed across studies (I²=0%). The overall effect was not statistically significant (Z=0.75; p-value =0.45). Figure 10 illustrates these statistically non-significant findings.

**Figure 11:**
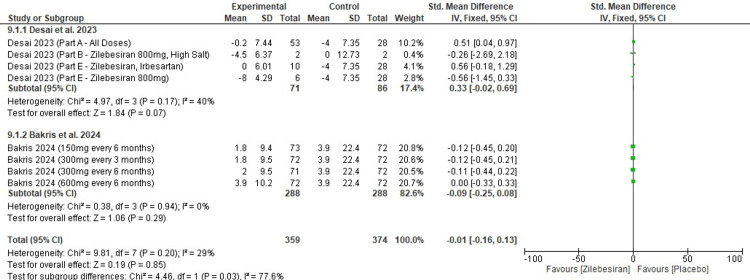
Forest plot for change from baseline in serum creatinine levels at 12 weeks [11,12]

Change in serum creatinine levels from baseline at 12 weeks: In the forest plot shown in Figure 11, the pooled SMD change in serum creatinine levels from baseline at 12 weeks was (SMD -0.01; 95% CI: -0.16, 0.13). This result was not statistically significant (p-value =0.20). A low degree of heterogeneity was observed across studies (I²=29%). In the subgroup analysis, individual studies also did not report any significant results, with all p-values remaining above 0.05.

**Figure 12:**
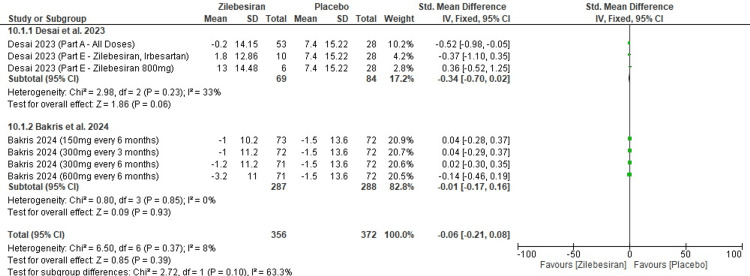
Forest plot for change from baseline in eGFR levels at 12 weeks eGFR: estimated glomerular filtration rate [11,12]

Change in mean eGFR levels from baseline at 12 weeks: No significant change in eGFR levels from baseline at 12 weeks was observed when comparing Zilebesiran and placebo (SMD -0.06; 95% CI: -0.21 to 0.08; p-value =0.39). The forest plot shown in Figure 12 illustrates this finding, reporting low heterogeneity across studies (I²=8%). Subgroup analysis also revealed no statistically significant results, as all individual studies reported p-values above 0.05.

Publication Bias

Given that only three studies were included in this meta-analysis, a reliable assessment of publication bias was not possible. Commonly used methods for evaluating publication bias, such as funnel plots and statistical tests, are not considered reliable when fewer than 10 studies are available, as they lack the statistical power to detect asymmetry or small-study effects [15].

Discussion

This meta-analysis reveals that Zilebesiran is associated with statistically significant and clinically meaningful reductions in 24-hour ambulatory mean office systolic and diastolic blood pressures at 12 and 24 weeks when compared to placebo, indicating a strong and sustained antihypertensive effect. These effects were observed in all the studies that were included in this meta-analysis. Zilebesiran consistently demonstrates a favourable effect, indicating a substantial decrease in blood pressure. The overall effect size is statistically significant, supporting the efficacy of Zilebesiran as a treatment option for hypertension. Greater reductions were observed in trials evaluating Zilebesiran as monotherapy. Although Zilebesiran was associated with an increased risk of total adverse events, no significant difference was observed in the incidence of severe adverse events. Additionally, no significant changes were observed in serum creatinine or eGFR levels at 12 weeks, suggesting that Zilebesiran does not adversely impact renal function acutely. The overall results support the efficacy of Zilebesiran in lowering blood pressure with an acceptable safety profile, reinforcing its potential as a long-acting option for the management of hypertension. However, the relatively short follow-up duration limits conclusions regarding long-term renal safety, particularly in high-risk groups such as patients with CKD, diabetes, and elderly populations. Further research is needed to fully understand and identify the most effective treatment regimens, which lie beyond the scope of this review and meta-analysis.

Zilebesiran is not just superior to other antihypertensives for the improvement in blood pressure that is seen, but it is also important to note that it only needs to be administered after three to six months subcutaneously. This means that patients do not have to take the drug every single day, which increases their compliance. It should be clarified that improved compliance is inferred from the extended dosing schedule rather than demonstrated directly in RCTs. On the other hand, it is important to note that this regimen might not be for everyone, as the drug is administered subcutaneously, so individuals with needle phobia would be reluctant to opt for this therapy. Consideration of cost effectiveness is also important. Long-acting RNAi therapy may reduce pill burden and potentially improve adherence-related outcomes, but the higher acquisition cost and need for subcutaneous delivery could limit widespread adoption [11].

The findings of this meta-analysis align closely with those presented by Maheta et al. (2025) [16], particularly in confirming the statistical and clinical significance of Zilebesiran in reducing blood pressure. Zilebesiran has a unique mechanism of action that targets hepatic angiotensinogen production through RNA interference therapy, consistently associated with meaningful reductions in blood pressure readings when administered every three to six months. This meta-analysis reported a statistically significant increase in the total number of adverse events compared to placebo, which was not highlighted in early phase studies; however, we found no significant difference in severe adverse events, which aligns with the favourable safety profile described by Maheta et al. (2025) [16]. A notable difference is that their review discusses the therapeutic potential of Zilebesiran in both essential and resistant hypertension, whereas this study focuses on the drug’s general efficacy and safety, without differentiating between subtypes of hypertension [16].

Menezes Junior et al. (2025) [17], exploring RNAi-based therapies targeting angiotensinogen for hypertension, report findings consistent with this study. Their scoping review demonstrated dose-dependent and sustained blood pressure reductions lasting up to six months after a single dose, highlighting the potential to improve adherence and maintain consistent RAAS suppression. However, this study also raises concerns regarding the potential disruption of RAAS-mediated compensatory mechanisms during hemodynamic stress. Given the long-acting effect of Zilebesiran, managing acute events with volume depletion like haemorrhage, sepsis, and anaphylaxis might be more difficult, placing them at a higher risk of refractory hypotension. This emphasises the need for reversal agents and careful use in acutely unwell patients. Future studies should evaluate potential monitoring strategies, such as biomarkers of RAAS suppression and explore possible contraindications or reversal agents to mitigate risk during acute illness. This meta-analysis was not designed to assess these risks and adverse outcomes, which remain important areas for future investigation [17].

The results of our meta-analysis align with current literature, including the review by Siddiqui et al. (2024) [18], which highlights the long-lasting antihypertensive effects and favourable safety profile of Zilebesiran. All included RCTs in our meta-analysis showed that Zilebesiran can substantially reduce blood pressure both as monotherapy and when used with other anti-hypertensive drugs; however, a greater reduction was seen with monotherapy. This is supported by the KARDIA-1 trial, as noted in both our analysis and the review by Siddiqui et al. (2024) [18], where monotherapy yielded more significant improvements than combination therapy. This highlights the importance of designing direct comparisons against standard antihypertensives to clarify how Zilebesiran should be integrated into clinical practice. The reduced response seen with co-administered agents may be due to drug interactions or external factors such as a high salt diet. These findings underscore the need for further research to better understand drug interactions and to determine the most effective combination strategies [18].

When considering the safety profile of Zilebesiran, it is important to note that even though it was associated with a higher incidence of adverse events compared to placebo, most of these were mild to moderate in nature. Serious adverse events reported were not found statistically significant between Zilebesiran and placebo. This shows that even though some individuals might consider this drug as having lower tolerability, its overall safety profile remains acceptable. Similarly, Flack (2025) [19] reports that critical questions remain regarding the long-term safety profile of Zilebesiran in patients with conditions such as chronic kidney disease or episodic hypotension, as well as the combination regimens with other antihypertensive drugs. These gaps highlight the need for further research, especially Phase 3 trials with extended follow-up and patient-level research to explore heterogeneity in response and safety according to baseline demographics and comorbidities, in order to establish safe and effective ways to use Zilebesiran in a diverse range of patients [19].

A previous meta-analysis published in early 2025 assessed the efficacy and safety of Zilebesiran using data from two RCTs and reported a significant reduction in blood pressure with a favourable safety profile [5]. However, it was limited by a smaller sample size and a short follow-up period of only three months. Our meta-analysis addresses these limitations by incorporating a third, more recent RCT, which increases the overall sample size and allows for the evaluation of both primary and secondary outcomes at both three and six months. Additionally, we conducted subgroup analyses and found that greater reductions in blood pressure occurred when Zilebesiran was used as monotherapy; an important finding not reported in the earlier review. Nonetheless, some heterogeneity persisted, likely related to differences in dosing regimens and background therapies, further highlighting the need for future research to clarify differential responses. These findings enhance the clinical relevance and strength of our study, offering a more comprehensive understanding of Zilebesiran in the management of hypertension [5].

The included studies underwent assessment for quality and publication bias, and all were identified as high-quality RCTs. These trials were conducted in various countries, providing a diverse geographic representation that strengthens the credibility of our findings. Each step of the meta-analysis was carried out independently by multiple reviewers to minimise bias and improve accuracy. This approach helped ensure that data extraction and interpretation were consistent and reliable. The overall quality of included RCTs and the structured approach of this review provide a strong basis for drawing evidence-based conclusions regarding the efficacy and safety of Zilebesiran.

Despite the strengths, our meta-analysis has several limitations. In some studies, only limited variables were reported, and certain values had to be extracted from graphs with large-scale calibrations, potentially introducing minor inaccuracies. In a few instances, standard deviation (SD) values were calculated from confidence intervals, which may have introduced variability. This meta-analysis was based on study-level rather than individual patient-level data, which limits the ability to account for differences in demographic characteristics and clinical presentations. A high degree of heterogeneity was observed, possibly due to variations in dosing regimens and background medication.

Furthermore, the relatively small sample size across included studies may also limit the statistical power of our analysis. It is important to consider that there could be a selection bias that could impact the results of this review. Only RCTs published in peer-reviewed journals were included; this leaves out studies in grey literature or other study designs that could provide alternate results showing decreased efficacy and increased adverse effects associated with this drug. Publication bias also remains a concern, as studies with null or negative findings may be underrepresented due to the limitation of this study in only including RCTs; this could lead to overestimation of efficacy and underreporting of adverse effects, especially given the role of industry sponsorship in most present-day trials.

Several studies showed heterogeneity in their outcomes, especially where Zilebesiran was used alongside other antihypertensive drugs. This shows that further research needs to be done with a larger sample size to establish whether Zilebesiran is sufficient as monotherapy or whether it should be combined with other drugs to optimally control blood pressure. If Zilebesiran needs to be combined with other drugs, then appropriate combinations to limit drug interactions need to be established. It is also important to note that there are limited RCTs that have been done on this drug, so it is hard to understand the efficacy and adverse effects of Zilebesiran in diverse populations, including those with comorbidities such as diabetes, chronic kidney disease, or obesity.

As the number of high-quality RCTs and sample sizes is limited, this shows that further research and trials are needed to establish the efficacy of this drug and the adverse effects that are associated with it. Additionally, this meta-analysis pooled data from various dosing regimens without distinguishing by dose or background therapy, limiting direct evaluation of dose-response relationships. As Zilebesiran shows promise with low adverse effects and progresses to Phase 3 trials, this would provide a larger dataset with more diverse populations and help establish whether this drug could provide a better alternative to the existing drugs for hypertension.

## Conclusions

This systematic review and meta-analysis demonstrate that Zilebesiran significantly reduces both ambulatory and office systolic and diastolic blood pressure at 12 and 24 weeks. Current evidence suggests a generally favourable renal and overall safety profile; however, this conclusion is limited by the short duration of follow-up and the need for long-term safety data, particularly in special populations such as patients with chronic kidney disease, diabetes, and the elderly. The findings of this study suggest that Zilebesiran has the potential to improve the management of hypertension compared to existing regimens, as it provides sustained control of blood pressure and enhances compliance due to infrequent dosing. However, current evidence is limited by a relatively small sample size and a lack of diversity among study populations. Therefore, this drug requires further large-scale research with a greater sample size and in more diverse populations. Future research should also focus on long-term cardiovascular outcomes beyond blood pressure reduction, patient-reported outcomes, including quality of life, and evaluation of the effects of Zilebesiran in individuals with complex comorbidities to establish its place in routine clinical practice.
